# Aortic Intima-Media Thickness and Aortic Diameter in Small for Gestational Age and Growth Restricted Fetuses

**DOI:** 10.1371/journal.pone.0126842

**Published:** 2015-05-27

**Authors:** M. Dolores Gomez-Roig, Edurne Mazarico, Esther Valladares, Laura Guirado, Mireia Fernandez-Arias, Antonio Vela

**Affiliations:** 1 Department of Obstetrics and Gynecology, Sant Joan de Déu University Hospital, Barcelona, Spain; 2 SAMID Network (Spanish Collaborative and Child Health Research Network), Madrid, Spain; Medical Faculty, Otto-von-Guericke University Magdeburg, Medical Faculty, GERMANY

## Abstract

**Objective:**

The objective of this study is to measure aortic intima-media thickness (aIMT) and aortic diameter (AD) in appropriate for gestational age (AGA) fetuses, small for gestational age (SGA) fetuses, and intrauterine growth restricted (IUGR) fetuses.

**Methods:**

Case-control study performed between June 2011 and June 2012. Forty-nine AGA fetuses, 40 SGA fetuses, and 35 IUGR fetuses underwent concomitant measurement of aIMT and AD at a mean gestational age of 34.4 weeks.

**Results:**

Median aIMT was higher in fetuses with IUGR (0.504 mm [95%CI: 0.477-0.530 mm]), than in SGA fetuses (0.466 mm [95% CI: 0.447–0.485 mm]), and AGA fetuses (0.471 mm [95% CI: 0.454-0.488 mm]) (p = 0.023). Mean AD was significantly lower in fetuses with IUGR (4.451 mm [95% CI: 4.258–4.655 mm]), than in AGA fetuses (4.74 mm [95% CI: 4.63-4.843 mm]) (p = 0.028).

**Conclusions:**

Growth restricted fetuses have a thicker aortic wall than AGA and SGA fetuses, which possibly represents preclinical atherosclerosis and a predisposition to later cardiovascular disease.

## Introduction

Cardiovascular disease (CVD) is a leading cause of death [1], and its pathological basis is arterial damage resulting in the development of arteriosclerosis. Arteriosclerosis describes diffuse thickening and stiffening of both the intima and media of large- and medium-size arteries. Atherosclerosis is a form of arteriosclerosis characterized by focal lesions in the intima of large- and medium-size arteries.

Originally, atherosclerosis was believed to arise only in adulthood. Recent studies, however, have demonstrated that although the clinical complications of atherosclerosis occur in adult life, the process of atherogenesis begins as a young child [2] or even as a fetus [3]. Cosmi et al [4,5] have shown that fetuses and children with intrauterine growth restriction (IUGR) have a greater aortic intima-media thickness (aIMT) than appropriate for gestational age (AGA) fetuses. Furthermore, low birthweight, caused by preterm birth or IUGR, is associated with a thicker aIMT and increased rates of CVD that are similar to those seen with major environmental risk factors, such as cigarette smoking, hypertension [6], hypercholesterolemia, and diabetes [7].

Theories proposing a fetal origin of atherosclerosis suggest that vascular lesions arise as a fetal adaptation to a state of malnutrition, such as IUGR. These adaptive processes may be cardiovascular, metabolic, or endocrine and may permanently alter structure and physiology [8,9,10]. The mechanisms placing IUGR fetuses at increased cardiovascular risk have not been clarified. Theoretical explanations include increased blood pressure, dyslipidemia, or reduced insulin-like growth factor [11]. There remains, however, no direct evidence that the changes observed in the structure of the aortic wall in fetuses and children are the precursors of arterial plaques, although they share the same physical sites [12]. Nevertheless, early identification of those infants at increased risk of atherosclerosis should allow for timely interventions that may delay or prevent the onset of cardiovascular events in adulthood.

The aim of this study is to measure aIMT and aortic diameter (AD) in AGA fetuses, small for gestational age (SGA) fetuses, and IUGR fetuses and compare the values between these groups. The primary purposes of the study are to determine whether aIMT and AD differ between IUGR fetuses and AGA fetuses and to examine whether aIMT and AD differ between SGA and IUGR fetuses. The latter comparison will evaluate whether these potential cardiovascular risk factors are present in all small fetuses or only those with placental insufficiency.

## Material and Methods

This case-control study was conducted at the *Sant Joan de Déu* University Hospital, Barcelona, from June 2011 to June 2012. Written informed consent was obtained from each patient before undergoing any study-related procedures. The study protocol was approved by the Institutional Review Board of Barcelona University Hospital, and there were no conflicts of interest.

Study participants were recruited after a routine third trimester fetal ultrasound was obtained. The inclusion criteria were singleton pregnancies and parental willingness to participate in the study. The exclusion criteria were a lack of consent, multiple pregnancies, congenital anomalies, diabetes, hypercholesterolemia, chronic hypertension, gestational hypertension, preeclampsia, thyroid or adrenal pathology, alcohol use, medication use (e.g. ritodrine), or clinical chorioamnionitis. Corticosteroids were permitted when administered for lung maturation only.

Ultrasound examinations were performed at a mean gestational age of 34.4 weeks (range, 32.1–35.4 weeks). All parameters were measured using a Siemens linear ultrasound probe S 2000 9L4 3.5-5MHz. Fetal biometries were measured and the estimated fetal weight was determined, followed by Doppler velocimetry of the umbilical artery, middle cerebral artery, cerebro-placental ratio and ductus venous (only in fetuses with an estimated fetal weight below the 10^th^ percentile). The fetus was then classified as AGA, SGA, or IUGR according to the following definitions:

1. **AGA**: fetuses with an estimated fetal weight between the 10^th^ and 90^th^ percentiles and normal Doppler velocimetry (umbilical artery pulsatility index, middle cerebral artery pulsatily index and cerebro-placental ratio).
2. **SGA**: fetuses with an estimated fetal weight below the 10^th^ percentile and normal Doppler velocimetry.
3. **IUGR**: fetuses with an estimated fetal weight below the 10^th^ percentile and an umbilical artery pulsatility index >95^th^ percentile.

**Fig 1:**
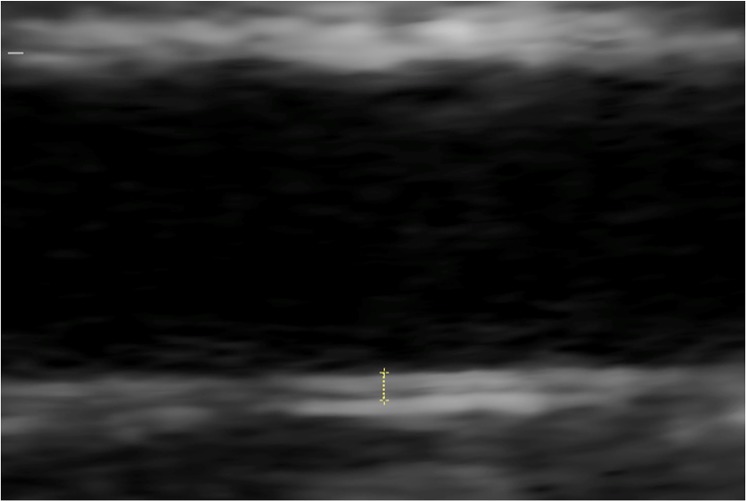
Measurement of aortic intima media thickness(AIMT).

**Fig 2:**
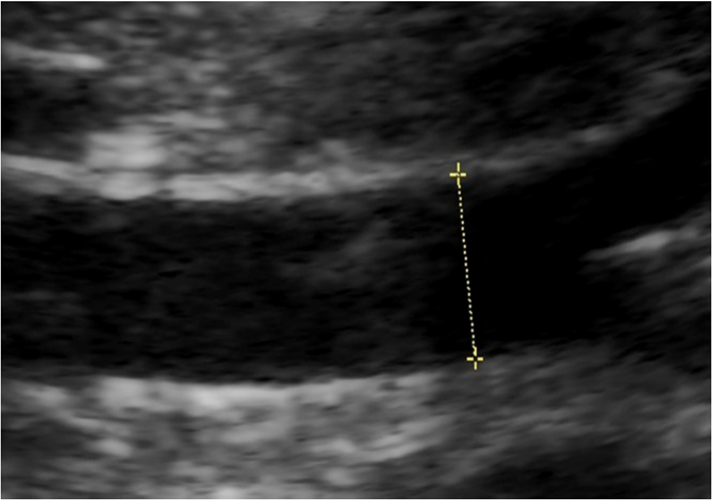
Measurement of aortic diameter (AD).

Measurements of aIMT and AD were obtained in the fetal abdominal aorta between the renal arteries and iliac arteries. Coronal and sagittal projections of the fetus were used to localize and subsequently magnify the area of the vessel to be measured. The image was frozen at the end of the systole to minimize variability. AIMT, defined as the distance between blood-intima interface and media-adventitia interface (Fig 1), and AD, defined as the distance between blood-intima interface at both ends of the vessel (Fig 2), were measured in this image. Each measurement was performed three times and the arithmetic mean was calculated. Fetal examination was easy but an experimented sonographer was required to perform it.

Intraobserver and interobserver reliability were calculated for the aIMT and AD measurements. Each measurement was obtained three times by a single observer (EV) during the initial ultrasound study. The measurements were repeated in a blinded manner by the original observer. After this, the images were unfrozen and new ones were obtained by a second observer. The second observer also repeated the measurements three times. The observers were not blinded to the fetus’ group. Both observers had advanced experience in fetal ultrasound. We performed an Interclass Correlation Coefficient (ICC) for absolute agreement. In addition to the fetal measurements, the maternal and fetal medical history, maternal and paternal anthropometric data, and other ultrasound and neonatal data were compiled. After calculating intraobserver and interobserver variability for the aIMT and AD measurements, the first observer measurements were used for the main data analyzed in the study. Statistical analysis was performed using the statistical package SPSS Statistics 19 (SPSS Inc. Chicago 12). Power analysis calculations indicated the need for a sample size of 33 patients in each group to detect significant differences for the primary endpoint: differences greater than 0.056 mm for aIMT and greater than 0.400 mm for AD between the IUGR, SGA, and AGA fetuses. This sample size calculation was based on an α = 0.05 and a β = 0.2. Normal distribution was tested using a combination of techniques, including application of the Kolmogorov-Smirnov test, histogram analysis, coefficient of skewness, and kurtosis. For normal distributions, comparisons were performed using the Student's t test for independent samples if there were two groups. For more than two groups, analysis of variance (ANOVA) and multiple analysis of variance (MANCOVA, adjusting by estimated fetal weight) were used. For non-normal distributions, nonparametric tests were used: the Mann-Whitney U test for comparisons of two groups and the Kruskal-Wallis H test for more than two groups. Statistical significance was defined as a P value <0.05. P values were adjusted using *post-hoc* tests for ANOVA and Kruskal-Wallis.

## Results

A total of 124 subjects were recruited for the study. They were divided as follows: 49 AGA fetuses, 40 SGA fetuses, and 35 IUGR fetuses. Maternal anthropometric, fetal anthropometric, and neonatal data are presented in Tables 1, 2, and 3, respectively. The number of IUGR fetuses in Table 3 is 33 instead of 35 because two patients delivered in another hospital, so the neonatal data could not be collected.

**Table 1:** Maternal anthropometric data.

MATERNAL DATA	AGA (n = 49)	SGA (n = 40)	IUGR (n = 35)	P value
Age (years)	32±5	29±6	31±6	0.152
Gestational age when measure is performed (weeks)	34.26±1.77	34.98±2.17	33.88±2.61	0.083
Maternal smoking	6 (12.24%)	6(15%)	7(20%)	0.634
Heigh (cm)	163.1±6.710	159.75±6.846	160.29±5.712	0.026
BMI before pregnancy	22±2.75	21.92±2.97	22.08±3.68	0.974
Birthweight (g)	3274.81±471.50	3011±582.05	3050.09±691.21	0.741

**Table 2:** Fetal anthropometric data.

FETAL DATA	AGA (n = 49)	SGA (n = 40)	IUGR (n = 35)	P value	P value adjusted^a^
Abdominal circumference (mm)	297.26±34.69	286.85±19.34	263.31±23.10	<0.001	
Estimated Fetal Weight (g)	2418.26±333.10	1930.98±429.36	1733±406.66	<0.001	
Weight Percentile	48.6±25.9	6.7±2	4.4±8.1	<0.001	
AIMT (mm)	0.471 (95% CI: 0.454–0.488)	0.466 (95%CI: 0.477–0.485)	0.504 (95%CI: 0.477–0.530)	0.022	0.023
Diameter of abdominal aorta (mm)	4.737 (95% CI: 4.630–4.843)	4.689 (95%CI: 4.524–4.854)	4.451 (95%CI: 4.258–4.645)	0.023	0.028

^a^ Adjusted by Estimated Fetal Weight

**Table 3:** Neonatal data.

FETAL DATA	AGA (n = 49)	SGA (n = 40)	IUGR (n = 33)	P value
Mode of delivery				0.107
Spontaneous delivery	29(59.18%)	27(67.5%)	16(48.48%)	
Forceps delivery	7(14.29%)	6(15%)	2(6.06%)	
Cesarean	13(26.53%)	7(17.5%)	15(42.86%)	
Gestational age at delivery (weeks)	39.59±1.92	38.57±1.55	37.06±2.40	<0.001
Birthweight (g)	3275.91±466.54	2590±382.71	2250.62±481.52	<0.001
Birth Weight percentile	52.3±19,3	7.1±2	3.4±6.8	<0.001
Arterial pH	7.24±0.05	7.25±0.07	7.24±0.07	0.639
Venous pH	7.31±0.05	7.29±0.07	7.29±0.06	0.072
Neonates gender				<0.001
Male	24(48.98%)	11(27.5%)	24(72.73%)	
Female	25(51.02%)	29(72.5%)	9(27.27%)	

Among IUGR fetuses, 9 of them had a pathological fetal median cerebral artery and 14 of them a pathological cerebro-placental ratio.

There were no statistically significant differences between groups for the maternal age, gestational age when measurements were performed, maternal smoking, body mass index prior to the pregnancy, and maternal birthweight. The maternal height was significantly higher for AGA fetuses than SGA and IUGR fetuses (p = 0.026). The IUGR fetuses were delivered at a significantly earlier gestational age than the SGA and AGA fetuses (p<0.001) because we induce labor in IUGR fetuses at 37 weeks. However, this does not affect our measurements, as they were obtained at a mean gestational age of 34.4 weeks.

**Fig 3:**
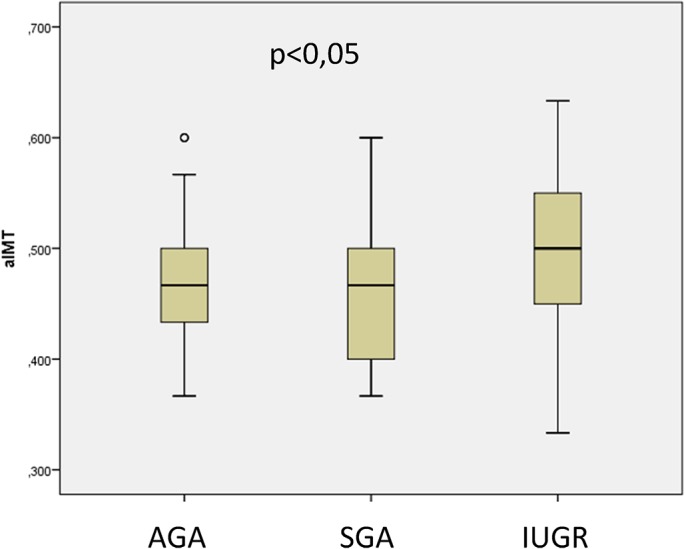
Difference in aortic intima media thickness measurements (mm) between fetuses appropiate for gestational age (AGA), small for gestational age (SGA) and with intrauterine growth restriction (IUGR).

**Fig 4:**
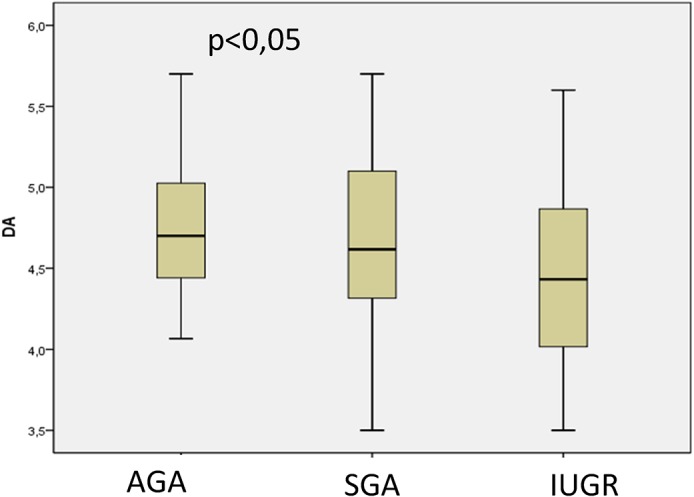
Difference in aortic diameter (AD) measurements (mm) between fetuses appropiate for gestational age (AGA), small for gestational age (SGA) and with intrauterine growth restriction (IUGR).

AD was normally distributed, but aIMT was not normally distributed; the results are presented as mean and 95% confidence intervals were also evaluated. The median aIMT was significantly greater in IUGR fetuses (0.504 mm [95% CI: 0.477–0.530 mm]), than AGA (0.471 mm [95% CI: 0.454–0.488 mm]) and SGA fetuses (0.466 mm [95% CI: 0.447–0.485 mm]) (p = 0.023) (p = 0.022) (Fig 3). After performing *post-hoc* tests for the Kruskal-Wallis analysis, the following p values were obtained: AGA vs. SGA, p = 0,05; AGA vs. IUGR, p = 0.045; and SGA vs. IUGR, p = 0.033. The mean AD was significantly thinner in IUGR fetuses (4.451 mm [95% CI: 4.258–4.655 mm]) than in AGA fetuses (4.74 mm [95% CI: 4.63–4.843 mm]) (p = 0.028) (Fig 4). After *post-hoc* tests were performed for ANOVA, the following p values were obtained: AGA vs. SGA, p = 0,1.00; AGA vs. IUGR, p = 0.025; and SGA vs. IUGR, p = 0.102.

Fetal gender was not uniformly distributed in the different groups (p<0.001), so the effect of gender on aIMT and AD measurements was studied. The median aIMT was 0.470 mm (0.370–0.630 mm) in females and 0.500 mm (0.330–0.600 mm) in males. The mean AD was 4.570 mm (4.126–5.014 mm) in females and 4.720 mm (4.195–5.245 mm) in males. These aIMT and AD values were not significantly different between male and female fetuses (p = 0.469 and 0.116, respectively).

Analysis of variance (ANOVA) adjusting for fetal gender and smoking were performed and the differences of AD and aIMT between AGA, IUGR and SGA fetuses were still significant (p = 0.003 and p = 0.03).

For aIMT, the intraobserver and interobserver correlations were 0.813 (0.727–0.875) and 0.713 (0.425–0.857), respectively (p <0.001 for both). For AD, the intraobserver and interobserver correlations were 0.985 (0.978–0.989) and 0.899 (0.799–0.949), respectively (p <0.001 for both).

## Discussion

The present study demonstrates that the aortic wall is thicker in fetuses with IUGR than AGA and SGA fetuses. These results are in agreement with previously published studies that correlate growth restriction and endothelial damage. For example, Skilton et al. [11] found that the maximum aIMT was higher in babies who had IUGR than in those with normal growth. Barker [7] found that low birthweight was an important and unrecognized risk factor for increased cardiovascular risk in adult life [13]. Barker proposed that the endocrine-metabolic reprogramming that enabled the growth restricted fetus to compensate for the hostile intrauterine environment might lead to metabolic syndrome in later life. Metabolic syndrome is characterized by the development of hypertension, hypercholesterolemia, impaired glucose tolerance and ischemic heart disease [7].

Almost all of the heretofore-published evidence arises from studies of neonates or adults. Only one previous study, reported by Cosmi et al [4], was conducted in the fetal population. This study examined aIMT in IUGR and AGA fetuses and in neonates after a mean follow-up of 18 months. By contrast, our study examined an additional group of patients: the SGA group. SGA fetuses have an estimated fetal weight below the 10^th^ percentile for gestational age, which is similar to IUGR fetuses, but they also have a normal Doppler, similar to AGA fetuses.

We found no differences in aIMT between AGA and SGA fetuses, although the aIMT was higher in IUGR fetuses than SGA fetuses. This finding suggests that the increase in aIMT is not present in all small fetuses, but only in those with Doppler abnormalities, which is a sign of adaptation to placental insufficiency. However, as contrast, some recent studies [14] showed that IMT values were significantly increased in SGA, with and without signs of severity, as compared with controls and these SGA newborns showed a trend for higher values of blood pressure.

It has been previously proposed that the increase in aIMT in IUGR fetuses is a compensatory reaction to an increased AD caused by a rise in intraluminal pressure [4,6]. Resultant transverse tears in the intima caused by pressure damage allow lipids to invade the intima and thereby increase the aIMT.

In contrast to this theory, our study did not demonstrate an increase in AD in IUGR fetuses. Indeed, AD was significantly lower in IUGR fetuses than AGA fetuses. This suggests that the increase in aIMT in our population was not a secondary response to an increase in AD. The increase in aIMT without an increase in AD decreased the arterial lumen significantly, contributing to a worsening of endothelial dysfunction and predisposing to adverse cardiovascular events.

Limitations of present study are the small number of patients included, data on maternal uterine artery Doppler was not collected, aIMT was measured manually and not using a computerized program, and there is not a postnatal follow up of patients. However, the interest of the results is high, and further studies with a wide number of patients and postnatal follow up are needed to establish the usefulness of these parameters as early markers of children at risk for cardiovascular disease and to study the mechanism of fetal programming of adult cardiovascular disease, which has not been elucidate yet.

In conclusion, we found that IUGR was associated with aortic wall thickening, suggesting that prenatal events and placental insufficiency may predispose to an increased cardiovascular risk. These cardiovascular changes were not present in fetuses that were constitutionally small or those with normal Doppler findings. An early identification of individuals with an increased risk of atherosclerosis could facilitate early application of appropriate health programs, which may help prevent adult CVD.
